# Adult-Onset Still’s Disease: An Atypical Presentation Refractory to Standard Treatment

**DOI:** 10.7759/cureus.64369

**Published:** 2024-07-11

**Authors:** Melody Esmaeili-Ghahfarokhi, Brandon H Kim, Farshid Bozorgnia

**Affiliations:** 1 Internal Medicine, University of California Irvine Medical Center, Orange, USA; 2 Rheumatology, University of California Irvine Medical Center, Orange, USA

**Keywords:** parvovirus b19, adult-onset still’s disease (aosd), immune response, macrophage activation syndrome (mas), hemophagocytic lymphohistiocytosis (hlh)

## Abstract

A previously healthy young female of Southeast Asian descent presented with a two-week history of polyarthritis, urticarial rash, sore throat, and 8.6 kg of unintentional weight loss. The initial workup revealed a positive parvovirus B19 polymerase chain reaction with hyperferritinemia. The patient was diagnosed with adult-onset Still’s disease (AOSD) secondary to parvovirus B19 infection. Bone marrow biopsy also showed evidence of hemophagocytic lymphohistiocytosis. Viral and bacterial infections may trigger AOSD in genetically susceptible hosts either via an unknown mechanism or by direct cytotoxic effect. This case shows an atypical presentation of AOSD, as well as the challenge in diagnosing and treating AOSD complicated by macrophage activation syndrome refractory to standard treatment.

## Introduction

Adult-onset Still’s disease (AOSD) is a rare multisystem inflammatory disorder that typically presents with inflammatory arthritis, quotidian fevers, and an evanescent macular rash. Viral and bacterial infections may trigger AOSD in genetically susceptible hosts either via an unknown mechanism or by direct cytotoxic effect. Macrophage activation syndrome is characterized by excessive immune activation and accumulation of macrophages with hemophagocytic activity, in essence, hemophagocytic lymphohistiocytosis (HLH) in the presence of inflammatory disorders [1]. Our case presents one of few cases of AOSD preceded by parvovirus B19 and with urticaria rather than the typical evanescent macular rash. Furthermore, we present an example of AOSD complicated by macrophage activation syndrome (MAS) that was refractory to standard treatment and discuss the possible mechanisms and approaches to treatment and earlier diagnosis.

## Case presentation

A 37-year-old female with no significant past medical history presented to the hospital with a two-week history of polyarthritis, urticarial rash, sore throat, and 8.6 kg of unintentional weight loss. Before admission, she visited an urgent care facility for her symptoms and was prescribed a four-day course of prednisone. Her condition, however, worsened.

Upon admission, the patient’s vitals were notable for a temperature of 101.8°F and a heart rate of 106 beats per minute. On physical examination, the patient had a macular and urticarial rash on her chest and forearms along with synovitis of the hands and wrists.

X-rays of the hands, knees, and feet revealed soft tissue swelling with no signs of erosions. General laboratory tests performed at the time of admission, as indicated in Table 1, showed leukocytosis, hyperferritinemia, and positive parvovirus B19 polymerase chain reaction PCR and IgG.

**Table 1:** Laboratory tests on admission. PCR = polymerase chain reaction

Laboratory parameters	Value	Reference range
White blood cell	30.3 K cells/µL	3.4–10.8 E3/µL
Hemoglobin	11.5 g/dL	11.5–15.0 g/dL
Ferritin	3,692 ng/mL	12–300 ng/mL
COVID-19	Negative	Negative
Parvovirus B19 PCR	Positive	Negative
Parvovirus B19 IgG	Positive	Negative
Parvovirus B19 IgM	Negative	Negative
Epstein-Barr virus IgG	Negative	Negative
Epstein-Barr virus IgM	Negative	Negative
Urinalysis	Normal	Normal

On hospital day four, ferritin was noted to increase to more than 15,000 ng/mL. The patient remained febrile to as high as 102.8°F. Her rash worsened, displaying the Koebner phenomenon and coinciding with her febrile episodes. A punch biopsy of the rash showed mild interstitial neutrophilic infiltrate alongside scattered necrotic keratinocytes. Therefore, based on the Yamaguchi and Cush criteria, the patient was diagnosed with AOSD as she had a quotidian fever, arthralgia, myalgia, polyarthritis, rash (although atypical), sore throat, negative antinuclear antibodies, elevated inflammatory markers, leukocytosis, and the development of transaminitis [2,3]. Therefore, she was started on Anakinra 100 mg daily.

**Figure 1:**
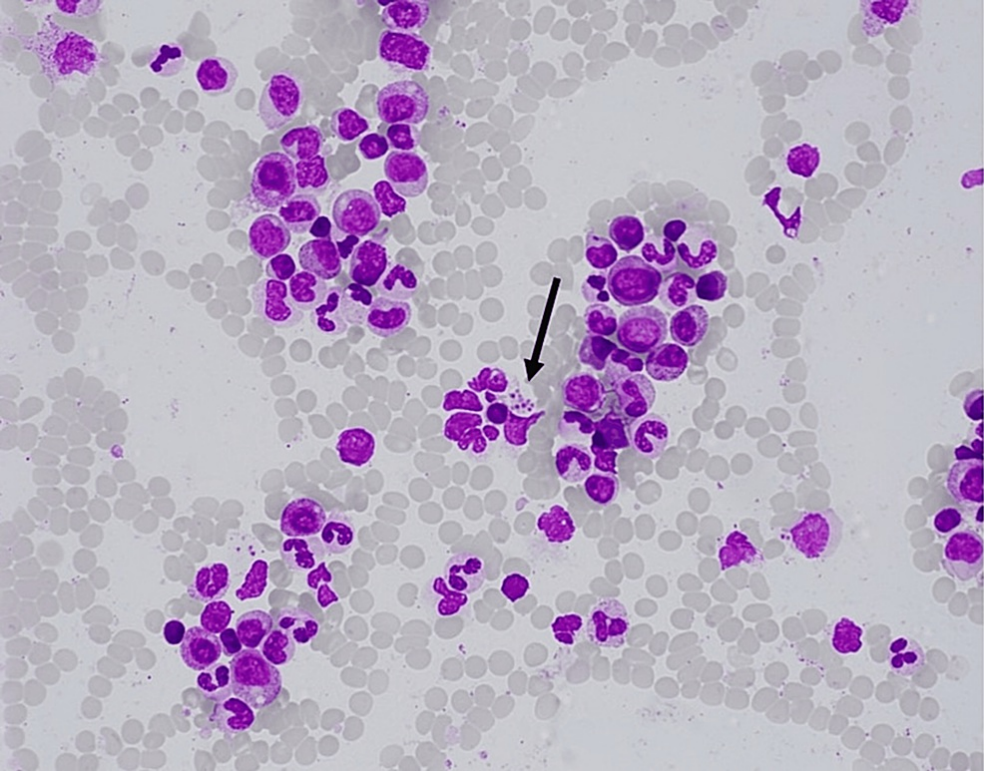
The bone marrow aspirate reveals a notable finding: hemophagocytic activity of platelets, indicated by the presence of histiocytes engulfing platelets (marked by the black arrow) under magnification at 40×. This observation underscores the pathologic hallmark of hemophagocytic lymphohistiocytosis, where excessive activation of the immune system leads to abnormal engulfment of blood cells by macrophages in the bone marrow.

**Figure 2:**
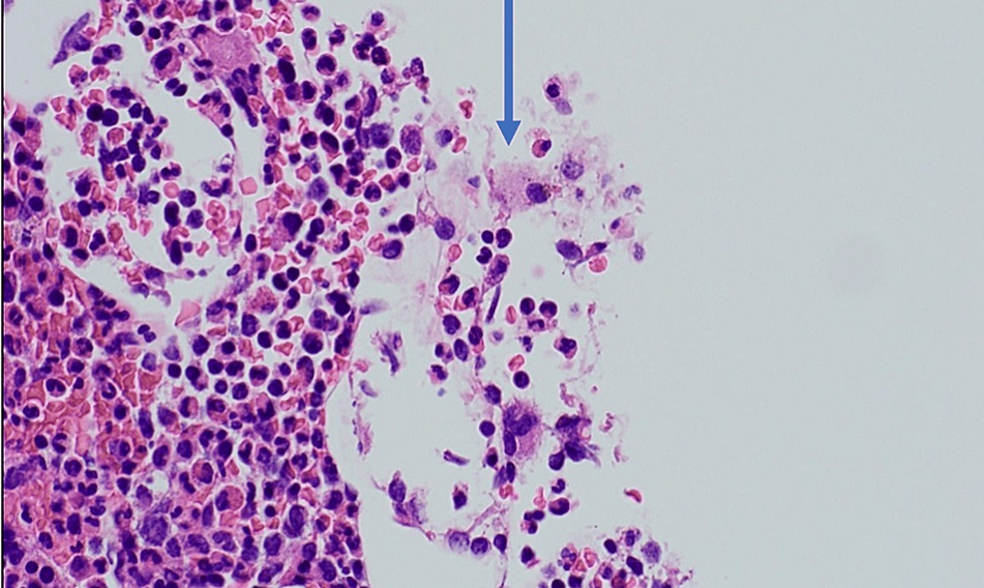
A notable increase in histiocytes is evident through the heightened expression of CD68 on immunohistochemical staining. This immunohistochemical marker serves to highlight the proliferation and activation of histiocytes, indicating the characteristic histopathological features associated with hemophagocytic lymphohistiocytosis.

Initially, she showed a favorable response to anakinra with a decline in ferritin levels. On hospital day 14, however, ferritin rebounded to more than 15,000 ng/mL. Anakinra was increased to 100 mg twice daily, accompanied by the initiation of intravenous methylprednisolone. Febrile episodes persisted and ferritin continued to increase, eventually to more than 60,000 ng/mL. She then developed hypertriglyceridemia and a worsening transaminitis. A bone marrow biopsy was performed which showed mild hemophagocytic activity detected by immunohistochemistry (Figures 1, 2). Given this, the patient was suspected to have HLH/MAS.

On hospital day 30, Anakinra was discontinued and ruxolitinib was started at 5 mg twice daily, eventually needing to be increased to 10 mg twice daily. At this time, cytomegalovirus PCR and IgG returned positive with negative IgM, and the patient was subsequently treated with gancyclovir as per Infectious Disease recommendations. Eventually, symptoms improved and the patient’s ferritin decreased. On hospital day 61, she was discharged on ruxolitinib and dexamethasone with outpatient follow-up.

## Discussion

AOSD is an uncommon systemic inflammatory disorder, with a prevalence of 1.6 in 1 million [4]. Although Still’s disease is a diagnosis of exclusion, specific diagnostic criteria help establish a diagnosis. The Yamaguchi criteria require five distinct features, including at least two major diagnostic criteria (such as a fever of at least 39°C lasting for a minimum of one week, arthralgias or arthritis persisting for two weeks or longer, a salmon-colored non-pruritic macular or maculopapular skin rash primarily appearing over the trunk or extremities during febrile episodes, and leukocytosis of 10,000/mL or greater with at least 80% granulocytes), as well as minor criteria (sore throat, lymphadenopathy, hepatomegaly or splenomegaly, abnormal liver function studies, particularly elevations in aspartate and alanine aminotransferase, lactate dehydrogenase levels, and negative results for antinuclear antibody and rheumatoid factor).

Quotidian fevers lasting for two weeks, maculopapular rash, arthralgia, leukocytosis, sore throat, and negative infectious and rheumatologic workup pointed toward Still’s disease, even though several aspects of our patient’s presentation were unusual. First, our patient’s positive parvovirus B19 IgG in the setting of a negative infectious workup suggests that parvovirus was likely the inciting factor. The identification of parvovirus B19-induced Still’s disease is not commonly described in the literature [5,6]. Furthermore, it is important to remember that not all Still’s patients present with classic evanescent rashes such as the case of our patient [7]. Our patient had urticaria which can be seen in Still’s disease, although less commonly [7].

Our patient also developed a potentially fatal complication of Still’s, MAS. MAS refers to HLH in association with inflammatory disorders and is characterized by excessive immune activation and accumulation of macrophages with hemophagocytic activity [1]. Lastly, and importantly, our patient worsened despite aggressive treatment with steroids and interleukin (IL)-1 inhibition. We speculate this may have been due to the initiation of treatment at a stage in the disease course where inflammation downstream of IL-1 had been activated in an exaggerated response.

The timing of treatment has become increasingly recognized as important in controlling AOSD and its progression. Early IL-6 and Janus kinase inhibition has been shown to be an important treatment modality for aggressive AOSD, while interferon-gamma antagonism is emerging as an important treatment target in AOSD patients with MAS [8,9]. Novel cytokine profiling methods for MAS offer the potential to improve patient outcomes by initiating earlier MAS treatment and management [9,10]. Elevated IL-18 levels in early MAS suggest that this cytokine may be a useful screening marker for MAS risk [1]. Interestingly, a newly established mechanistic connection between mTORC1 and inflammation in the context of Still’s disease and MAS suggests the potential use of mTORC1 inhibitors, such as rapamycin, as therapeutic interventions [11]. 

## Conclusions

Our patient with AOSD presented with several less common features, including a probable parvovirus trigger, a non-evanescent rash, and the emergence of MAS. Additionally, IL-1 inhibition did not completely ameliorate the patient’s condition once MAS had developed. Cytokine profiling, which our patient may have benefited from, is a promising avenue for possible interventions before the onset of overt MAS.
